# Why is noma a neglected-neglected tropical disease?

**DOI:** 10.1371/journal.pntd.0008435

**Published:** 2020-08-20

**Authors:** M. Leila Srour, Denise Baratti-Mayer

**Affiliations:** 1 Health Frontiers, Vientiane, Laos; 2 Institute of Global Health, University of Geneva, Geneva, Switzerland; University of Kelaniya, SRI LANKA

## Abstract

Noma is an orofacial gangrene affecting primarily children living in extreme poverty in remote parts of subtropical and tropical countries. Mortality and disability are high, and survivors often have physical and functional deformities resulting in stigma and isolation. Many healthcare professionals and primary healthcare workers where noma risk factors exist have no knowledge about noma and its implications. Public health measures to improve nutrition, immunizations, sanitation, and access to healthcare and measures to eliminate extreme poverty can lead to the eradication of noma. Research allocation has been insufficient to study the epidemiology, treatment, and prevention of noma. In a recent editorial by Hotez and colleagues in PLOS Neglected Tropical Diseases (NTDs), “What constitutes an NTD?” Noma is not included. The exclusion of noma from NTDs constitutes this preventable childhood disease as a neglected neglected disease. The purpose of this article is the inclusion of noma with the PLOS NTDs. Increased awareness and attention to noma can lead to the eradication of this disease affecting the world’s most vulnerable.

Noma (cancrum oris) is an orofacial gangrene, an opportunistic infection, affecting primarily chronically malnourished young children, living in extreme poverty and often without access to medical care [1]. Many medical professionals and front-line healthcare workers have no knowledge about noma and would not recognize the early stages when treatment reduces mortality or be able to refer a noma survivor [2,3]. Although noma is the quintessential neglected tropical disease (NTD), noma is not included in lists of NTDs and remains a neglected-neglected tropical disease. In the recent PLOS NTD editorial, “What constitutes a neglected tropical disease?,” noma is not included [4]. The purpose of this article is to support the inclusion of noma with Neglected Tropical Diseases, especially by PLOS NTDs. The integration of noma with NTDs could improve awareness about noma and target investments in research regarding its etiology, diagnosis, and treatment leading to prevention, early detection, and eradication of this preventable childhood disease that affects the world’s most vulnerable children.

The World Health Organization (WHO) has four criteria for inclusion in their list of NTDs. First, the disease is a cause of stigma, morbidity and mortality of impoverished people. Second, the disease primarily occurs in tropical or subtropical regions. Third, control and eradication are feasible with known public health strategies. Fourth, there has been insufficient research support to determine best treatment and control options. Noma meets all of the above criteria.

First, noma is primarily found in remote regions, affecting young children living in extreme poverty. Noma is called the “Face of Poverty” [5] because the disease severely damages the faces of children living in extreme poverty and uniquely illustrates the severity of children’s lives whose parents lack sufficient resources to adequately feed their children. Untreated mortality is high (80% to 90%), and survivors are often severely affected with facial deformities that cause difficulty with eating, speaking, and appearance, leading to stigmatization and isolation [1,5]. Survivors of noma are often discriminated against and unable to attend school, socialize, marry, or find employment [6].

Noma survivors often present after decades of suffering seeking surgical rehabilitation, having been denied access to education, vocation, marriage, and social acceptance. Surgical treatment of survivors is difficult, requires experienced surgical teams, and, usually, is not available to survivors of noma.

Second, noma is primarily reported from poor developing countries in Africa and Asia, but noma can occur wherever the risk factors exist. Risk factors for noma include extreme poverty, malnutrition, lack of exclusive breastfeeding, lack of access to medical care and immunizations, maternal undernutrition and grand multiparity, preceding febrile illness (especially measles or malaria), poor oral health, and reduced diversity of oral bacterial flora [1,7].

Third, public health interventions (such as promotion of exclusive breastfeeding), adequate complementary foods, sufficient vitamins, immunizations, sanitation, and access to healthcare result in the prevention, detection, and early treatment of noma and disease sequelae. The risk factors for noma are common to the current NTDs, which often exist where noma is found. Vertical programs to target noma alone may be ineffective, so including noma where it belongs with the diseases of poverty can break the cycle of neglect.

Fourth, research regarding the epidemiology, diagnosis, treatment, and prevention of noma has been insufficient to guide control and eradication. The funding and implementation of public health interventions could lead to the disappearance of noma [8]

## Noma disease burden

In 1998, WHO estimated a global incidence of 140,000 cases of noma and a prevalence of 770,000 survivors with severe sequelae [9]. An annual incidence of 30,000 to 40,000 may be a better estimate due to the methodology and challenge of estimating a disease that is rarely reported in the acute stages [8,10].

A Swiss Tropical and Public Health Institute group calculated disability adjusted life years (DALYs) using a simplified disease model, including weights for minor and major sequelae and death. Preliminary results from this Global Burden of Disease Study on noma, by Sophie Haesen and her colleagues, calculated 1 to 10 million DALYs lost due to noma. This is the first global effort to estimate noma’s impact [11]. The wide range is generated from uncertainties about incidence, mortality, and surgical rehabilitation. The 2010 Global Burden of Disease Study enabled comparison of the relative impact of NTDs. Estimated DALYs lost due to NTDs ranged between 0.14 and 3.32 million years. Even though the estimated burden of disease from noma is comparable, noma was not included [12].

## Why is noma neglected?

In 1649, noma was included in a textbook of neglected diseases by Arnoldus Bootius called “Observationes Medicae de Affectibus Omissis.” Noma affected young children in Europe and North America until the early 20th century, when public health progress and economic development led to the disappearance of noma [1] until its recurrence in concentration camps in World War II [13].

Nowadays noma affects the most impoverished children in remote areas where there are no records of births or deaths. Primary healthcare workers and traditional healers often do not recognize noma [3,14]. Noma is not taught in medical schools either in the occidental world or in the countries concerned. Noma survivors remain isolated by poverty and social stigma [6].

National neglect of noma results from the high mortality without documentation. Survivors remain hidden as estimates suggest only 10% to 15% of survivors seek care [6]. As noma is a “biological indicator of extreme poverty” [5], political and health authorities may prefer to ignore noma and what it signifies. No efforts may be made to find and document noma incidence and mortality.

The omission of noma from lists of NTDs results in international neglect due to lack of awareness and knowledge. Noma is noncommunicable, so it does not cause the alarms of contagious diseases or threaten developed countries. Further, noma is not a target for pharmaceutical companies, because the acute phase can be treated with inexpensive antibiotics.

In 2012, a study published by the United Nations Human Rights Council Advisory Committee, on severe malnutrition and childhood diseases with children affected by noma as an example, found that noma is evidence of the worst violations of basic human rights and should be addressed at a global level, with formal inclusion in the WHO list of NTDs [15].

We appeal to PLOS NTDs to include noma in their list of NTDs. In the last decade, PLOS NTDs has published seven significant articles with noma as the main subject. The inclusion of noma in the PLOS NTDs list will encourage increased awareness, enhance research, and promote the eradication of this neglected childhood disease. Noma survivors are a shocking reminder of the continual neglect of the world’s most vulnerable, who live in extreme poverty.
